# Common Data Elements for COVID-19 Neuroimaging: A GCS-NeuroCOVID Proposal

**DOI:** 10.1007/s12028-021-01192-6

**Published:** 2021-02-11

**Authors:** Brian L. Edlow, Melanie Boly, Sherry H.-Y. Chou, David Fischer, Daniel Kondziella, Lucia M. Li, Christine L. Mac Donald, Molly McNett, Virginia F. J. Newcombe, Robert D. Stevens, David K. Menon, GCS-NeuroCOVID

**Affiliations:** 1 Center for Neurotechnology and Neurorecovery, Department of Neurology, Massachusetts General Hospital and Harvard Medical School, 175 Cambridge Street – Suite 300, Boston, MA 02114 USA; 2 Athinoula A. Martinos Center for Biomedical Imaging, Massachusetts General Hospital, Charlestown, MA USA; 3 Departments of Neurology, University of Wisconsin, Madison, WI USA; 4 Wisconsin Institute for Sleep and Consciousness, Department of Psychiatry, University of Wisconsin-Madison, Madison, WI USA; 5 Departments of Critical Care Medicine, Neurology, and Neurosurgery, University of Pittsburgh School of Medicine, Safar Center for Resuscitation Research, Pittsburgh, PA USA; 6 Department of Neurology, Rigshospitalet, Copenhagen University Hospital, Copenhagen, Denmark; 7 Department of Clinical Medicine, University of Copenhagen, Copenhagen, Denmark; 8 UK Dementia Research Institute – Centre for Health Care and Technology, Imperial College London, London, UK; 9 Department of Neurological Surgery, University of Washington, Seattle, WA USA; 10 College of Nursing, The Ohio State University, Columbus, OH USA; 11 University Division of Anaesthesia, University of Cambridge, Addenbrooke’s Hospital Cambridge, Cambridge, UK; 12 Departments of Anesthesiology and Critical Care Medicine, Neurology, Neurosurgery, Johns Hopkins University School of Medicine, Baltimore, MD USA

## Introduction

Patients with coronavirus disease 2019 (COVID-19) present with a broad spectrum of neurological disorders whose features have been described in numerous reports since the beginning of the global pandemic [1–5]. The incidence and prevalence of these disorders range from 3.5% to 84% among patients with COVID-19 [2, 5, 6], with neurological symptoms ranging from mild (e.g., anosmia) to severe (e.g., encephalopathy, stroke) [1, 3]. A global community of researchers and clinicians has mobilized in an effort to elucidate the pathophysiological mechanisms underlying neurological injury in patients with COVID-19. These mechanisms include hypoxia [7], inflammation [3], hypercoagulability [3], endothelial infection by the severe acute respiratory syndrome coronavirus 2 (SARS-CoV-2) [8], autoimmunity, and possibly encephalitis due to direct viral infection of the central nervous system by SARS-CoV-2 [9]. However, fundamental questions about pathophysiology and mechanisms of recovery remain, hampering our ability to diagnose, prognosticate, and treat patients with neurological disorders associated with COVID-19.

In response to the urgent need to advance knowledge about the many neurological disorders associated with COVID-19, there is growing recognition that international scientific collaboration is essential [10–13]. A coordinated global effort can accelerate the pace of discovery in COVID-19-related neurological disorders, enabling timely translation into clinical practice. Accordingly, multiple large-scale studies were launched in the spring of 2020 to study COVID-19 neurological disorders, including the CoroNerve Study Group [4], the Italian Society of Neurology’s NEUROCOVID study [14], the James S. McDonnell Foundation’s COVID-19 Recovery of Consciousness Consortium [15], the European Academy of Neurology’s EANCore project [10, 16], the Latin American Brain Injury Consortium (LABIC) [17], the Prospective Observational Study of Subarachnoid hemorrhage and IntracereBral hemorrhage patients in Latin America (POSSIBLE) network’s COVID-19 study [17], and the Global Consortium Study of Neurological Dysfunction in COVID-19 (GCS-NeuroCOVID) [17, 18]. The EAN and the GCS-NeuroCOVID consortia have established a formal collaboration resulting in a global network that encompasses over 400 sites worldwide [12]. As part of that collaboration, the EAN and GCS-NeuroCOVID consortia have committed to harmonizing their common data elements (CDEs) and data definitions to the extent possible, as indicated in their joint publication [12]. Many of these networks and consortia are represented in the Forum on Neurology and COVID-19 by the Brain Health Unit of the World Health Organization, a collaborative network of international clinicians and scientists who participate in discussions about the clinical management, surveillance and research of COVID-19-related neurological manifestations and impact.

Central to these international efforts is the application of neuroimaging techniques, which play a key role in the diagnostic and prognostic evaluation of patients with COVID-19 neurological disorders and may also provide important insights into pathophysiological mechanisms. Neuroimaging can be challenging to perform in patients with COVID-19 because of safety precautions that are necessary to protect other patients and hospital staff. This challenge is greatest in patients with COVID-19 critical illness and acute respiratory distress syndrome (ARDS), many of whom are too unstable to travel to a computed tomography (CT) or magnetic resonance imaging (MRI) scanner. The first case report describing neuroimaging findings associated with severe COVID-19—in an adult patient who was comatose due to hemorrhagic leukoencephalitis [19]—was not published until March 31, 2020, more than four months after the first cases of COVID-19 were reported in Wuhan, China, highlighting the logistical challenges associated with COVID-19 neuroimaging.

As the global pandemic approaches the end of its first year, and as protocols for safe patient transport are more widely implemented, neuroimaging tests such as head CT and brain MRI are increasingly utilized in clinical care and in research studies of adult patients with neurological disorders from COVID-19, including in the acute, subacute and chronic phases. These studies have begun to reveal the structural and functional correlates of neurological disorders in patients with COVID-19 [3, 20, 21]. This is a topic of profound neuroscientific interest and clinical relevance, as evidenced by the more than 600 citations of the first COVID-19 neuroimaging case report in less than six months [19], and by the publication of numerous articles about COVID-19 neuroimaging during the pandemic’s first nine months [22, 23].

Development of harmonized and uniform data elements is now a key goal to facilitate the most useful data repositories for investigating neurological disorders in COVID-19 [13]. Experience with other neurological diseases has demonstrated the benefit of collecting data in a systematic and consistent way, an approach championed by the National Institutes of Health (NIH) CDEs process, which provides CDEs for a range of diseases (https://www.commondataelements.ninds.nih.gov/). In order to facilitate a similar process for patients with neurological disorders in the setting of COVID-19, the GCS-NeuroCOVID group consulted widely to construct consensus CDEs for neurological complications and sequelae of COVID-19. This CDE effort has three tiers of data collection. Tier 1 [17] is a minimal dataset, which typically fulfills registry-level data abstraction and is designed to avoid the need to collect identifiable patient data. Tier 2 represents the workhorse of data collection for global studies and provides a level of detail that would be available from institutions in most developed countries and many developing countries. Tier 3 represents even more specialized data collection by centers and investigators with expertise in a given area and would likely require custom-built data collection designed by the study group addressing a specific research question.

As part of the Tier 2 CDE development effort, GCS-NeuroCOVID convened a work group to create CDEs for neuroimaging studies of adult patients with COVID-19. Our goal was to create neuroimaging CDEs with the following characteristics:

1. Capable of capturing the broad spectrum of findings reported to date in adult patients with COVID-19;
2. Adaptable based on emerging evidence that might be reported in the future, particularly as more patients are imaged in the chronic phase; and
3. Feasible to implement in hospitals around the world.

Given the rapidly evolving landscape of COVID-19 neuroimaging, the CDEs that we report here are intended as a starting point for further efforts by members of the international medical and scientific community, who can adapt and refine these proposed COVID-19 neuroimaging CDEs as additional neuroimaging discoveries emerge. These CDEs should be collected in conjunction with other relevant GCS-NeuroCOVID CDEs characterizing clinical characteristics and outcomes in adult patients. Ultimately, we expect that these COVID-19 neuroimaging CDEs will evolve with ongoing efforts to standardize data acquisition and data sharing, with the goal of improving care and outcomes for patients with COVID-19 neurological disorders globally.

## Methods

### CDE Development Meetings

A nine-member Neuroimaging Work Group was convened as part of GCS-NeuroCOVID to develop neuroimaging CDEs for adult patients with COVID-19. The work group met weekly online from July 16 to October 8, 2020, with the goal of creating COVID-19 neuroimaging CDEs for Tier 2 of the GCS-NeuroCOVID project. Whereas Tier 1 of GCS-NeuroCOVID aimed to capture the frequency of neuroimaging in patients with COVID-19, Tier 2 aims to characterize relevant neuroimaging features.

Given that Tier 2 is planned as a multicenter, international study of data acquired as part of clinical practice, we developed the Tier 2 CDEs to capture data from commonly available techniques (e.g., head CT and conventional MRI), not from advanced imaging techniques such as functional MRI [21] or diffusion tensor imaging [24, 25]. CDEs for advanced neuroimaging techniques will be developed to support future GCS-NeuroCOVID Tier 3 studies conducted at centers that are able to prospectively acquire such data.

### Literature Review and Feature Classification

The GCS-NeuroCOVID Neuroimaging Work Group’s first task was to perform a comprehensive literature review of all studies published on COVID-19 neuroimaging in the adult patient population. We used the search terms “COVID-19”, “neuroimaging”, “computed tomography”, “CT”, “magnetic resonance imaging”, “MRI”, “CT Perfusion”, “MRI Perfusion”, “MRI spectroscopy”, “Positron Emission Tomography” and “PET” using the *PubMed* database and the *bioRxiv* and *medRxiv* preprint servers. These database searches, performed between July 16 and October 8, 2020, were supplemented by knowledge of manuscripts listed on authoritative websites (https://blogs.bmj.com/jnnp/2020/05/01/the-neurology-and-neuropsychiatry-of-covid-19/). Manuscript selection was based on consensus review to optimize the diversity of imaging features represented.

After compiling imaging reports described in published and preprint manuscripts, we organized data by imaging features, not by any presumed interpretive or diagnostic classification. For example, hypointensities detected on susceptibility-weighted imaging (SWI) were classified as such, not as microhemorrhages, given that these lesions can also represent microthrombi and/or air bubbles (e.g., in patients treated with extracorporeal membrane oxygenation). Similarly, hyperintense lesions on diffusion-weighted imaging (DWI) were classified as diffusion restriction (as long as there was corresponding hypointense signal on the apparent diffusion coefficient map), not as ischemic infarcts, given that these lesions can also be caused by seizures, brain tumors, abscesses and/or hypercellularity (e.g., in patients with infectious or para-infectious encephalitis). In short, we chose a feature-based approach to avoid making assumptions without firm evidence about the mechanisms by which COVID-19 affects the nervous system.

### Adaptation of Established CDEs for Neuroimaging of COVID-19

After completion of the literature review and classification of the described imaging features, we reviewed existing neuroimaging CDEs commissioned by the NIH (https://commondataelements.ninds.nih.gov). Our goal was to leverage these existing CDEs and, whenever possible, to use CDEs that were already defined according to established standards. These previously published CDEs provide the benefit of user familiarity and prior vetting by neuroimaging experts [26–28].

Consistent with previously published CDEs, we organized the COVID-19 neuroimaging CDEs into case report forms (CRFs) by imaging techniques. Techniques were eligible for inclusion based on prespecified criteria: (1) routine acquisition of the technique in clinical practice; and (2) at least one publication describing the clinical application of the technique in patients with COVID-19.

Importantly, most previously published neuroimaging CDEs describe specific diagnostic syndromes (e.g., ischemic stroke, intracerebral hemorrhage). Thus, although our overall goal was to use a feature-based approach to COVID-19 neuroimaging CDEs, we include previously published CDEs in “Syndromic CDEs” sections of the CRFs. Our rationale for including these “Syndromic CDEs” sections is that the emerging literature on COVID-19 neurological disorders includes descriptions of patients with well-characterized neurological syndromes, such as ischemic stroke [3], even if the mechanistic association between the syndromes and COVID-19 is unclear.

### Proposal for New Feature-Based COVID-19 Neuroimaging CDEs

For the neuroimaging features described in COVID-19 publications that were not accounted for by previously published CDEs, we created new descriptive CDEs based on consensus opinion. These descriptive CDEs are listed in separate sections titled “Feature-based CDEs” within the CRFs. By organizing the COVID-19 neuroimaging CDEs into complementary “Syndromic CDEs” and “Feature-based CDEs” sections, we aimed to provide investigators with the flexibility to thoroughly characterize all neuroimaging findings, regardless of whether they can fit within previously described neurological syndromes.

### Classifying the Pathophysiologic Association of Imaging Findings with COVID-19

We also provide investigators with an opportunity to enter data about presumed mechanisms of neurological injury and their relatedness to COVID-19. At the end of each “Syndromic CDEs” section and each “Feature-based CDEs” section of a CRF, we created new CDEs pertaining to the presumed cause of the imaging findings. Specifically, we provide investigators with an opportunity to indicate whether the findings are associated with COVID-19. Such data will enable assessment of the international community’s interpretation of newly observed imaging findings, while also providing hypothesis-generating data for future studies.

### CDE Classification as Core or Supplemental

All CDEs were classified as “core” or “supplemental” based on the consensus opinion of the Work Group. Given that the goal of the Tier 2 GCS-NeuroCOVID study is to identify common neuroimaging features in patients with neurological disorders related to COVID-19, we assigned the “core” designation to all CDEs that documented the presence or absence of those features. We assigned the “supplemental” designation to CDEs that characterize the neuroanatomic distribution and chronicity of imaging features, details which may aid neuroscientific and translational research. Limiting the number of core CDEs was intended to reduce the burden of data entry, which can lead to incomplete CRFs and reduced participation in multicenter international trials. This trade-off between thoroughness and feasibility is particularly relevant during the COVID-19 pandemic, when many clinicians and researchers are strained for time and resources.

## Results

### Adaptation of Previously Proposed CDEs to COVID-19

The neuroimaging CDEs previously proposed by the NIH that were most relevant to COVID-19 included those developed for ischemic stroke [27], traumatic brain injury [26] and subarachnoid hemorrhage [28]. Based on these previously developed CDEs, we created six CRFs, each representing a neuroimaging technique: (1) head CT; (2) conventional MRI; (3) CT perfusion; (4) MR perfusion; (5) MR spectroscopy; and (6) positron emission tomography (PET). An overview of the six CRFs is provided in Table 1. Of note, we limited the PET CDEs to studies obtained in standard clinical practice, such as FDG- and ^15^O-PET. CDEs for PET studies utilizing additional radioligands will be added in Tier 3. Given limited prior reports on neuroimaging studies utilizing cerebral angiography [29] and spinal cord imaging [30], CDEs for these modalities will also be added in Tier 3.

**Table 1:** Organization of common data elements into case report forms

	CT	MRI	Perfusion CT/MR	MR spectroscopy	PET
Patient Information	Time since symptom onset, time since COVID-19 diagnosis				
Clinical Indication	Scan purpose, neurological symptoms at time of scan				
Technical Information	Scanner, sequences				
Imaging Result	Normal, abnormal (acute), abnormal (chronic), abnormal (acute and chronic), indeterminate				
Syndromic CDEs	Stroke, hemorrhage, hydrocephalus				
Feature-based CDEs	Signal characteristics			Metabolites	Tracer uptake
Mechanism	Associated, not associated, or uncertain association with COVID-19				

The COVID-19 neuroimaging common data elements (CDEs) proposed for Tier 2 of the GCS-NeuroCOVID study are organized into six case report forms, each representing an imaging modality (columns): head computed tomography (CT), conventional magnetic resonance imaging (MRI), CT perfusion, magnetic resonance (MR) perfusion, MR spectroscopy, and positron emission tomography (PET). Within each case report form, CDEs are organized into sections (rows)

### Neuroimaging Features in Adult Patients with Neurological Disorders Related to COVID-19

The complete list of neuroimaging features that we identified as of October 8, 2020 are provided in Table 2 and listed in the “Feature-based CDEs” sections of the six CRFs that we release with this manuscript.

**Table 2:** Feature-based Common Data Elements

Case report form	Feature-based CDEs
CT	Hypodensity, hyperdensity, enhancement
MRI	Decreased volume, increased volume, diffusion restriction, T2-weighted hyperintensity, susceptibility-weighted or T2*-weighted hypointensity, enhancement, cystic changes
CT Perfusion	CBF, MTT, Time to peak, or TMax abnormalities
MR Perfusion	
MR Spectroscopy	Metabolite abnormalities: N-acetyl- aspartate, Choline, Creatine, Myo-inositol, Lactate, Glutamine/glutamate
PET	Localization of tracer uptake

*CBF* cerebral blood flow, *CDE* common data element, *CT* computed tomography, *MTT* mean transit time, *MRI* magnetic resonance imaging, *TMax* time to maximum

### Dissemination of CDEs for COVID-19 Neuroimaging

We release version 1.0 of the proposed neuroimaging CDEs for adult patients with COVID-19 as a set of six CRFs (https://zenodo.org/record/4404855). For each CRF, we created an abbreviated version that contains core CDEs and a comprehensive version that contains core and supplemental CDEs.

We encourage feedback regarding modifications to the CDEs, which can be submitted via email to the corresponding author. We are committed to an adaptive approach based on emerging evidence, with rapid distribution of modifications in real time using online scientific portals.

## Discussion

As the international community looks toward the next stage of the COVID-19 pandemic, there is growing recognition that global multicenter collaboration, data standardization and data sharing are essential to advance knowledge and improve care for patients with neurological disorders caused by COVID-19. To support this effort, we developed and now disseminate proposed CDEs for COVID-19 neuroimaging of adult patients. We designed these CDEs to be widely accessible and easy to implement at both academic medical centers and community hospitals. The COVID-19 neuroimaging CDEs leverage previous CDE efforts supported by the NIH, ensuring consistency with established standards for data acquisition. Newly proposed CDEs specific to COVID-19 were added based on a review of COVID-19 neuroimaging studies published during the first 10 months of the global pandemic. All COVID-19 CDEs, organized in 6 technique-specific CRFs, are available at https://zenodo.org/record/4404855.

The CDEs proposed here are feature-based, a strategy intended to avoid presumptions about the mechanisms by which COVID-19 affects the nervous system. At this stage of the pandemic, given an incomplete understanding of disease pathophysiology, we believe that neuroimaging data are most likely to enable knowledge creation if they are categorized using CDEs that are descriptive and not diagnostic in nature. Diagnostic categorizations can then be proposed, and revised, as more becomes known about how SARS-CoV-2 affects the nervous system. We therefore envision this CDE development effort as a dynamic process with rapid revisions reflecting ongoing progress in the field of COVID-19 neuroimaging. Only with a comprehensive, global commitment to data standardization and data sharing can the international community advance knowledge and improve care for patients with neurological disorders associated with COVID-19.

## Supplementary Information

Below is the link to the electronic supplementary material.

Supplementary material 1 (DOCX 34 kb)

Supplementary material 2 (DOCX 26 kb)

Supplementary material 3 (DOCX 67 kb)

Supplementary material 4 (DOCX 35 kb)

Supplementary material 5 (DOCX 87 kb)

Supplementary material 6 (DOCX 38 kb)

Supplementary material 7 (DOCX 30 kb)

Supplementary material 8 (DOCX 23 kb)

Supplementary material 9 (DOCX 24 kb)

Supplementary material 10 (DOCX 22 kb)

Supplementary material 11 (DOCX 31 kb)

Supplementary material 12 (DOCX 23 kb)

## Data Availability

This work was supported by the NIH Director’s Office (DP2HD101400), NIH National Institute for Neurological Disorders and Stroke (R01NS091618, R21NS113037, R25NS06574309), National Center for Advancing Translational Sciences (UL1 TR001857), Addenbrooke’s Charitable Trust (COVID-19: understanding its long-term respiratory, cardiac and neuro-psychiatric sequelae and the determinants of adverse outcomes), United Kingdom Research and Innovation (The COVID-19: Clinical Neuroscience Study [COVID-CNS]), the National Institute of Health Research (NIHR), the Johns Hopkins University Discovery and StAAR awards, the University of Pittsburgh Dean’s Faculty Advancement Award, James S. McDonnell Foundation’s COVID-19 Recovery of Consciousness Consortium, the Lundbeck Foundation (R349-2020–658), and the Academy of Medical Sciences/The Health Foundation Clinician Scientist Fellowship.
