# Supplementary material for: Common Data Elements for COVID-19 Neuroimaging: A GCS-NeuroCOVID Proposal
Source: Neurocrit Care. 2021 Feb 11;34(2):365–70. doi: 10.1007/s12028-021-01192-6 (PMC7878171; doi:10.1007/s12028-021-01192-6)
Supplement: Supplementary file 1 — Supplementary material 1 (DOCX 34 kb) [file 12028_2021_1192_MOESM1_ESM.docx]

## User Guide

- This Case Report Form (CRF) is designed to capture the broad spectrum of imaging findings detected by computed tomography (CT) perfusion in patients with COVID-19.
- The CRF is organized into five sections, each of which contains a set of Common Data Elements (CDEs):
  - Patient Information
  - Clinical Indication
  - Technical Information
  - Result
  - Feature-based CDEs
- With respect to the laterality of imaging findings, please report the findings in anatomic convention, not radiologic convention.
- With respect to the chronicity of imaging findings, please use your clinical judgment. Acute findings are those which are thought to be recent. If a finding has both acute and chronic components, please indicate both.
- Core CDEs, which are required for completion of this CRF, are indicated with an asterisk (*). All other CDEs are Supplemental.

## PATIENT INFORMATION

1. Study ID number:* ____
2. Date of onset of first symptom of COVID-19 (MM / DD / YYYY):* ____ / ____ / ______
3. Date of onset of first neurological symptom (MM / DD / YYYY):* ____ / ____ / ______
4. Date of first positive test for SARS-CoV-2 (MM / DD / YYYY):* ____ / ____ / ______
5. Date of imaging study (MM / DD / YYYY):* ____ / ____ / ______

## CLINICAL INDICATION

1. Scan purpose (select all that apply):*

Diagnostic

Post-treatment

Monitoring

Follow-up

Other, specify: __________

1. Neurological symptoms at time of scan (select all that apply):*

None

Focal deficits

Seizures

Confusion/delirium

Coma/disorder of consciousness

Other: ____________________

## TECHNICAL INFORMATION

- 1. CT details:

1. Number of slices:

64

128

256

320

Other, specify: ____

1. Slice thickness (mm): _____
2. Study technically satisfactory:

Yes No Unknown

## RESULT*

Normal Abnormal (acute) Abnormal (chronic) Abnormal (acute and chronic) Indeterminate

FEATURE-BASED CDEs

1) Localization of perfusion abnormalities:

Global Regional (if regional, fill in table below)

2) If global:

a. Chronicity:

Acute

Chronic

Uncertain

b. Measurement type:

CBF

MTT

Time to peak

TMax

3) If regional:

**Brain Region Table**

| **Brain Region** | **Laterality and Chronicity** | | **Measurement (check all**  **that apply)** |
| --- | --- | --- | --- |
|  | **Right** | **Left** |  |
| Frontal lobe | Acute  Chronic  Uncertain | Acute  Chronic  Uncertain | CBF  MTT  Time to peak  TMax |
| Parietal lobe | Acute  Chronic  Uncertain | Acute  Chronic  Uncertain | CBF  MTT  Time to peak  TMax |
| Temporal lobe | Acute  Chronic  Uncertain | Acute  Chronic  Uncertain | CBF  MTT  Time to peak  TMax |
| Occipital lobe | Acute  Chronic  Uncertain | Acute  Chronic  Uncertain | CBF  MTT  Time to peak  TMax |
| Insula | Acute  Chronic  Uncertain | Acute  Chronic  Uncertain | CBF  MTT  Time to peak  TMax |
| Cerebellum | Acute  Chronic  Uncertain | Acute  Chronic  Uncertain | CBF  MTT  Time to peak  TMax |
| Midbrain | Acute  Chronic  Uncertain | Acute  Chronic  Uncertain | CBF  MTT  Time to peak  TMax |
| Pons | Acute  Chronic  Uncertain | Acute  Chronic  Uncertain | CBF  MTT  Time to peak  TMax |
| Medulla | Acute  Chronic  Uncertain | Acute  Chronic  Uncertain | CBF  MTT  Time to peak  TMax |
| Corona radiata | Acute  Chronic  Uncertain | Acute  Chronic  Uncertain | CBF  MTT  Time to peak  TMax |
| Periventricular | Acute  Chronic  Uncertain | Acute  Chronic  Uncertain | CBF  MTT  Time to peak  TMax |
| Corpus Callosum | Acute  Chronic  Uncertain | Acute  Chronic  Uncertain | CBF  MTT  Time to peak  TMax |
| Anterior limb  internal capsule | Acute  Chronic  Uncertain | Acute  Chronic  Uncertain | CBF  MTT  Time to peak  TMax |
| Posterior limb  internal capsule | Acute  Chronic  Uncertain | Acute  Chronic  Uncertain | CBF  MTT  Time to peak  TMax |
| Caudate | Acute  Chronic  Uncertain | Acute  Chronic  Uncertain | CBF  MTT  Time to peak  TMax |
| Globus Pallidus | Acute  Chronic  Uncertain | Acute  Chronic  Uncertain | CBF  MTT  Time to peak  TMax |
| Putamen | Acute  Chronic  Uncertain | Acute  Chronic  Uncertain | CBF  MTT  Time to peak  TMax |
| Thalamus | Acute  Chronic  Uncertain | Acute  Chronic  Uncertain | CBF  MTT  Time to peak  TMax |

Mechanism

1. Presumed etiolog(ies) of feature-based imaging finding(s) (check all that apply):*

Hypoxia

Hypoxic-ischemic injury

Traumatic brain injury

Inflammation/encephalitis

Related to extracorporeal membrane oxygenation (ECMO)

Hypoglycemia

Abscess

Tumor

Seizure

Other: _________________

1. Presumed association of feature-based finding(s) with COVID-19:*

Associated

Not associated

Uncertain
