# Supplementary material for: Common Data Elements for COVID-19 Neuroimaging: A GCS-NeuroCOVID Proposal
Source: Neurocrit Care. 2021 Feb 11;34(2):365–70. doi: 10.1007/s12028-021-01192-6 (PMC7878171; doi:10.1007/s12028-021-01192-6)
Supplement: Supplementary file 3 — Supplementary material 3 (DOCX 67 kb) [file 12028_2021_1192_MOESM3_ESM.docx]

## User Guide

- This Case Report Form (CRF) is designed to capture the broad spectrum of imaging findings detected by head computed tomography (CT) in patients with COVID-19.
- The CRF is organized into seven sections, each of which contains a set of Common Data Elements (CDEs):
  - Patient Information
  - Clinical Indication
  - Technical Information
  - Result
  - Syndromic CDEs
  - Feature-based CDEs
  - Appendix
- If an imaging finding is consistent with a well-established syndrome, please describe the finding using the Syndromic CDEs section and not the Feature-based CDEs section.
- If an imaging finding is not consistent with a well-established syndrome, or if there is uncertainty about the interpretation of the finding, please describe the finding using the Feature-based CDEs section and not the Syndromic CDEs section.
- With respect to the laterality of imaging findings, please report the findings in anatomic convention, not radiologic convention.
- With respect to the chronicity of imaging findings, please use your clinical judgment. Acute findings are those which are thought to be recent. If a finding has both acute and chronic components, please indicate both.
- For imaging grading scales, references are provided in an appendix at the end of the CRF.
- Core CDEs, which are required for completion of this CRF, are indicated with an asterisk (*). All other CDEs are Supplemental.

## PATIENT INFORMATION

1. Study ID number:* ____
2. Date of onset of first symptom of COVID-19 (MM / DD / YYYY):* ____ / ____ / ______
3. Date of onset of first neurological symptom (MM / DD / YYYY):* ____ / ____ / ______
4. Date of first positive test for SARS-CoV-2 (MM / DD / YYYY):* ____ / ____ / ______
5. Date of imaging study (MM / DD / YYYY):* ____ / ____ / ______

## CLINICAL INDICATION

1. Scan purpose (select all that apply):*

Diagnostic

Post-treatment

Monitoring

Follow-up

Other, specify: __________

1. Neurological symptoms at time of scan (select all that apply):*

None

Focal deficits

Seizures

Confusion/delirium

Coma/disorder of consciousness

Other: ____________________

## TECHNICAL INFORMATION

1. Imaging modality (select all that apply):*

Non-contrast head CT: CT angiogram neck:

CT venogram head: Postcontrast head CT:

CT angiogram head:

1. CT acquisition parameters:
   - 1. Scanner number of slices: _____
     2. slice thickness: _____ mm
2. Imaging scanner manufacturer name (choose one):

Agfa Hologic

Carestream GE

Hitachi Konica Minolta

Other

## RESULT*

Normal Abnormal (acute) Abnormal (chronic) Abnormal (acute and chronic) Indeterminate

SYNDROMIC CDEs

Ischemic Stroke

1. Present?:*

Yes  No  Indeterminate

1. If yes, location and chronicity of infarct(s) (select all that apply):

Brain Region Table

| **Brain Region** | **Laterality and Chronicity** | |
| --- | --- | --- |
|  | **Right** | **Left** |
| Frontal lobe | Acute  Chronic  Uncertain | Acute  Chronic  Uncertain |
| Parietal lobe | Acute  Chronic  Uncertain | Acute  Chronic  Uncertain |
| Temporal lobe | Acute  Chronic  Uncertain | Acute  Chronic  Uncertain |
| Occipital lobe | Acute  Chronic  Uncertain | Acute  Chronic  Uncertain |
| Insula | Acute  Chronic  Uncertain | Acute  Chronic  Uncertain |
| Cerebellum | Acute  Chronic  Uncertain | Acute  Chronic  Uncertain |
| Midbrain | Acute  Chronic  Uncertain | Acute  Chronic  Uncertain |
| Pons | Acute  Chronic  Uncertain | Acute  Chronic  Uncertain |
| Medulla | Acute  Chronic  Uncertain | Acute  Chronic  Uncertain |
| Corona radiata | Acute  Chronic  Uncertain | Acute  Chronic  Uncertain |
| Periventricular | Acute  Chronic  Uncertain | Acute  Chronic  Uncertain |
| Corpus Callosum | Acute  Chronic  Uncertain | Acute  Chronic  Uncertain |
| Anterior limb  internal capsule | Acute  Chronic  Uncertain | Acute  Chronic  Uncertain |
| Posterior limb  internal capsule | Acute  Chronic  Uncertain | Acute  Chronic  Uncertain |
| Caudate | Acute  Chronic  Uncertain | Acute  Chronic  Uncertain |
| Globus Pallidus | Acute  Chronic  Uncertain | Acute  Chronic  Uncertain |
| Putamen | Acute  Chronic  Uncertain | Acute  Chronic  Uncertain |
| Thalamus | Acute  Chronic  Uncertain | Acute  Chronic  Uncertain |

1. Hemorrhagic Transformation:

Present?:*

Yes  No  Indeterminate

1. Additional findings (select all that apply):

Malignant edema

Hyperdense artery, specify:

Hyperdense venous sinus or cortical vein, specify: _________

1. Leukoaraiosis (i.e. small vessel ischemic disease, white matter hypodensities) on CT:
   1. Present?:*

Yes  No  Indeterminate

- 1. White matter hypodensity grading:

Van Swieetten Scale^1^:

- - 1. Anterior white matter:

No lesion

Lesions partly involving the white matter

Lesions extending up to the subcortical region

- - 1. Posterior white matter:

No lesion

Lesions partly involving the white matter

Lesions extending up to the subcortical region

Hemorrhage

1. Intraparenchymal hematoma:
   1. Present?:*

Yes  No  Indeterminate

- 1. Intracerebral hemorrhage (ICH) volume^2^ (ABC/2): ______
  2. Perihematomal edema volume (cc): ______

**Lobar Region Table**

| Lobar Region | Laterality and Chronicity | |
| --- | --- | --- |
|  | Right | Left |
| Frontal | Acute  Chronic  Uncertain | Acute  Chronic  Uncertain |
| Parietal | Acute  Chronic  Uncertain | Acute  Chronic  Uncertain |
| Temporal | Acute  Chronic  Uncertain | Acute  Chronic  Uncertain |
| Occipital | Acute  Chronic  Uncertain | Acute  Chronic  Uncertain |

**Deep Region Table**

| Deep Region | Laterality and Chronicity | |
| --- | --- | --- |
|  | Right | Left |
| Caudate | Acute  Chronic  Uncertain | Acute  Chronic  Uncertain |
| Putamen | Acute  Chronic  Uncertain | Acute  Chronic  Uncertain |
| Globus Pallidus | Acute  Chronic  Uncertain | Acute  Chronic  Uncertain |
| Thalamus | Acute  Chronic  Uncertain | Acute  Chronic  Uncertain |
| Cerebellum | Acute  Chronic  Uncertain | Acute  Chronic  Uncertain |
| Midbrain | Acute  Chronic  Uncertain | Acute  Chronic  Uncertain |
| Pons | Acute  Chronic  Uncertain | Acute  Chronic  Uncertain |
| Medulla | Acute  Chronic  Uncertain | Acute  Chronic  Uncertain |

1. Intraventricular hemorrhage (IVH):
   1. Present?*

Yes  No  Indeterminate

- 1. Graeb IVH scale^3^:

Use the following for I – II:

1= Trace amount of blood or mild bleeding;

2 = < Half of the ventricle filled with blood;

3= > Half of the ventricle filled with blood;

4= Ventricle expanded and filled with blood

- - 1. Right lateral ventricle score:
    2. Left lateral ventricle score:

Use the following for III – IV:

1= Blood present without dilatation;

2= Ventricle expanded and filled with blood

- - 1. Third ventricle score:
    2. Fourth ventricle score:
    3. Total score (Sum of scores I. – IV.; maximum score is 12):
  1. Volume (cc): ______

1. Subarachnoid hemorrhage (SAH):
   1. Present?:*

Yes  No  Indeterminate

- 1. Modified Fisher scale^4^:

Grade 0: No SAH or IVH

Grade 1: SAH less than 1 mm thick, no IVH

Grade 2: SAH less than 1 mm thick, with IVH

Grade 3: SAH more than 1 mm thick, no IVH

Grade 4: SAH more than 1 mm thick, with IVH

1. Epidural Hematoma:
   1. Present?:*

Yes  No  Indeterminate

- 1. Volume: _____ cc
  2. Thickness: _______ mm
  3. Location:

Frontal  Right  Left

Parietal  Right  Left

Temporal  Right  Left

Occipital  Right  Left

Posterior fossa  Right  Left

1. Subdural Hematoma:
   1. Present?:*

Yes  No  Indeterminate

- 1. Thickness: ______ mm
  2. Location (check all that apply):

Frontal  R  L

Parietal  R  L

Temporal  R  L

Occipital  R  L

Interhemispheric

Tentorial  R  L

Posterior fossa  R  L

- 1. Subdural hematoma type:

Subacute  Acute  Chronic

Vascular Abnormalities

1. Present?:*

Yes  No  Indeterminate

1. If present, specify type of abnormality:

Intracranial arterial occlusion  Cervical arterial occlusion

Aneurysm  Arteriovenous malformation

Venous thrombosis  Cavernous malformation

Deep venous anomaly  Dural arterio-venous fistula

Mass Effect and Midline Shift:

1. Midline shift:
   1. Present?:*

Yes  No  Indeterminate

- 1. If present, specify (mm): _______
  2. If present, anatomic landmark for measurement

Pineal gland

Septum pellucidum

Other _____________

1. Effacement of basal cisterns?*

Open  Partially effaced  Completely effaced

1. Herniation:
   1. Present?:*

Yes  No  Indeterminate

- 1. Location:

Subfalcine

Uncal

Tonsillar

[Transcalvarial](https://www.google.com/search?client=firefox-b-1-d&q=transcalvarial+herniation&spell=1&sa=X&ved=0ahUKEwiMzejYz7bgAhWLiOAKHaJIAx0QkeECCCkoAA)

1. Hydrocephalus:
   1. Present?:*

Yes  No  Indeterminate

- 1. If yes, bicaudate index:
  2. Type of hydrocephalus (select all that apply)
     1. Communicating
     2. Non-communicating
     3. Ex vacuo
     4. Uncertain

1. If there is midline shift, effacement of basal cisterns, herniation, or hydrocephalus, what is the dominant lesion type causing the finding?

ischemic stroke

intraparenchymal hemorrhage

subarachnoid hemorrhage

subdural hemorrhage

epidural hemorrhage

other: ___________________

Mechanism

1. Presumed association of syndrome with COVID-19:*

Associated

Not associated

Uncertain

## FEATURE-BASED CDEs

1) Hypodensity not previously documented in syndromic CDE section

a. Present?:*

Yes  No  Indeterminate

b. Localization:

Global Regional (if regional, fill in table)

**Brain Region Table**

| **Brain Region** | **Laterality and Chronicity** | |
| --- | --- | --- |
|  | **Right** | **Left** |
| Frontal lobe | Acute  Chronic  Uncertain | Acute  Chronic  Uncertain |
| Parietal lobe | Acute  Chronic  Uncertain | Acute  Chronic  Uncertain |
| Temporal lobe | Acute  Chronic  Uncertain | Acute  Chronic  Uncertain |
| Occipital lobe | Acute  Chronic  Uncertain | Acute  Chronic  Uncertain |
| Insula | Acute  Chronic  Uncertain | Acute  Chronic  Uncertain |
| Cerebellum | Acute  Chronic  Uncertain | Acute  Chronic  Uncertain |
| Midbrain | Acute  Chronic  Uncertain | Acute  Chronic  Uncertain |
| Pons | Acute  Chronic  Uncertain | Acute  Chronic  Uncertain |
| Medulla | Acute  Chronic  Uncertain | Acute  Chronic  Uncertain |
| Corona radiata | Acute  Chronic  Uncertain | Acute  Chronic  Uncertain |
| Periventricular | Acute  Chronic  Uncertain | Acute  Chronic  Uncertain |
| Corpus Callosum | Acute  Chronic  Uncertain | Acute  Chronic  Uncertain |
| Anterior limb  internal capsule | Acute  Chronic  Uncertain | Acute  Chronic  Uncertain |
| Posterior limb  internal capsule | Acute  Chronic  Uncertain | Acute  Chronic  Uncertain |
| Caudate | Acute  Chronic  Uncertain | Acute  Chronic  Uncertain |
| Globus Pallidus | Acute  Chronic  Uncertain | Acute  Chronic  Uncertain |
| Putamen | Acute  Chronic  Uncertain | Acute  Chronic  Uncertain |
| Thalamus | Acute  Chronic  Uncertain | Acute  Chronic  Uncertain |

2) Hyperdensity not previously documented in syndromic CDE section

a. Present?:*

Yes  No  Indeterminate

b. Localization:

Global Regional (if regional, fill in table)

**Brain Region Table**

| **Brain Region** | **Laterality and Chronicity** | |
| --- | --- | --- |
|  | **Right** | **Left** |
| Frontal lobe | Acute  Chronic  Uncertain | Acute  Chronic  Uncertain |
| Parietal lobe | Acute  Chronic  Uncertain | Acute  Chronic  Uncertain |
| Temporal lobe | Acute  Chronic  Uncertain | Acute  Chronic  Uncertain |
| Occipital lobe | Acute  Chronic  Uncertain | Acute  Chronic  Uncertain |
| Insula | Acute  Chronic  Uncertain | Acute  Chronic  Uncertain |
| Cerebellum | Acute  Chronic  Uncertain | Acute  Chronic  Uncertain |
| Midbrain | Acute  Chronic  Uncertain | Acute  Chronic  Uncertain |
| Pons | Acute  Chronic  Uncertain | Acute  Chronic  Uncertain |
| Medulla | Acute  Chronic  Uncertain | Acute  Chronic  Uncertain |
| Corona radiata | Acute  Chronic  Uncertain | Acute  Chronic  Uncertain |
| Periventricular | Acute  Chronic  Uncertain | Acute  Chronic  Uncertain |
| Corpus Callosum | Acute  Chronic  Uncertain | Acute  Chronic  Uncertain |
| Anterior limb  internal capsule | Acute  Chronic  Uncertain | Acute  Chronic  Uncertain |
| Posterior limb  internal capsule | Acute  Chronic  Uncertain | Acute  Chronic  Uncertain |
| Caudate | Acute  Chronic  Uncertain | Acute  Chronic  Uncertain |
| Globus Pallidus | Acute  Chronic  Uncertain | Acute  Chronic  Uncertain |
| Putamen | Acute  Chronic  Uncertain | Acute  Chronic  Uncertain |
| Thalamus | Acute  Chronic  Uncertain | Acute  Chronic  Uncertain |

3) Enhancement not documented in syndromic CDE section

a. Present?:*

Yes  No  Indeterminate

b. Localization:

Global Regional (if regional, fill in table)

**Brain Region Table**

| **Brain Region** | **Laterality and Chronicity** | |
| --- | --- | --- |
|  | **Right** | **Left** |
| Frontal lobe | Acute  Chronic  Uncertain | Acute  Chronic  Uncertain |
| Parietal lobe | Acute  Chronic  Uncertain | Acute  Chronic  Uncertain |
| Temporal lobe | Acute  Chronic  Uncertain | Acute  Chronic  Uncertain |
| Occipital lobe | Acute  Chronic  Uncertain | Acute  Chronic  Uncertain |
| Insula | Acute  Chronic  Uncertain | Acute  Chronic  Uncertain |
| Cerebellum | Acute  Chronic  Uncertain | Acute  Chronic  Uncertain |
| Midbrain | Acute  Chronic  Uncertain | Acute  Chronic  Uncertain |
| Pons | Acute  Chronic  Uncertain | Acute  Chronic  Uncertain |
| Medulla | Acute  Chronic  Uncertain | Acute  Chronic  Uncertain |
| Corona radiata | Acute  Chronic  Uncertain | Acute  Chronic  Uncertain |
| Periventricular | Acute  Chronic  Uncertain | Acute  Chronic  Uncertain |
| Corpus Callosum | Acute  Chronic  Uncertain | Acute  Chronic  Uncertain |
| Anterior limb  internal capsule | Acute  Chronic  Uncertain | Acute  Chronic  Uncertain |
| Posterior limb  internal capsule | Acute  Chronic  Uncertain | Acute  Chronic  Uncertain |
| Caudate | Acute  Chronic  Uncertain | Acute  Chronic  Uncertain |
| Globus Pallidus | Acute  Chronic  Uncertain | Acute  Chronic  Uncertain |
| Putamen | Acute  Chronic  Uncertain | Acute  Chronic  Uncertain |
| Thalamus | Acute  Chronic  Uncertain | Acute  Chronic  Uncertain |

4) Arterial abnormality not previously documented in Syndromic CDE section:

a. Present?:*

Yes  No  Indeterminate

b. Localization:

Global Regional (if regional, fill in table)

**Arterial Table**

| Vessel | Laterality | Occlusion | Vessel Narrowing^ | Dissection | Other^^ |
| --- | --- | --- | --- | --- | --- |
| Cervical ICA | L |  |  |  |  |
|  | R |  |  |  |  |
| Intracranial ICA | L |  |  |  |  |
|  | R |  |  |  |  |
| MCA | L |  |  |  |  |
|  | R |  |  |  |  |
| ACA | L |  |  |  |  |
|  | R |  |  |  |  |
| Cervical vertebral | L |  |  |  |  |
|  | R |  |  |  |  |
| Intracranial vertebral | L |  |  |  |  |
|  | R |  |  |  |  |
| Basilar |  |  |  |  |  |
| PCA | L |  |  |  |  |
|  | R |  |  |  |  |
| Other (specify vessel):  ____________________ | L |  |  |  |  |
|  | R |  |  |  |  |

^ findings suggestive of stenosis, vasculitis or reversible cerebral vasoconstriction syndrome

^^ please specify here: _______________________________________________

Mechanism

1. Presumed etiolog(ies) of feature-based imaging finding(s) (check all that apply):*

Hypoxia

Hypoxic-ischemic injury

Traumatic brain injury

Inflammation/encephalitis

Related to extracorporeal membrane oxygenation (ECMO)

Hypoglycemia

Abscess

Tumor

Seizure

Other: _________________

1. Presumed association of feature-based finding(s) with COVID-19:*

Associated

Not associated

Uncertain

APPENDIX

1 van Swieten, J. C., Hijdra, A., Koudstaal, P. J. & van Gijn, J. Grading white matter lesions on CT and MRI: a simple scale. *J Neurol Neurosurg Psychiatry* **53**, 1080-1083, (1990).

2 Kothari, R. U. *et al.* The ABCs of measuring intracerebral hemorrhage volumes. *Stroke* **27**, 1304-1305, (1996).

3 Morgan, T. C. *et al.* The Modified Graeb Score: an enhanced tool for intraventricular hemorrhage measurement and prediction of functional outcome. *Stroke* **44**, 635-641, (2013).

4 Frontera, J. A. *et al.* Prediction of symptomatic vasospasm after subarachnoid hemorrhage: the modified fisher scale. *Neurosurgery* **59**, 21-27; discussion 21-27, (2006).
