# Supplementary material for: Common Data Elements for COVID-19 Neuroimaging: A GCS-NeuroCOVID Proposal
Source: Neurocrit Care. 2021 Feb 11;34(2):365–70. doi: 10.1007/s12028-021-01192-6 (PMC7878171; doi:10.1007/s12028-021-01192-6)
Supplement: Supplementary file 4 — Supplementary material 4 (DOCX 35 kb) [file 12028_2021_1192_MOESM4_ESM.docx]

## User Guide

- This Case Report Form (CRF) is designed to capture the broad spectrum of imaging findings detected by head computed tomography (CT) in patients with COVID-19.
- The CRF is organized into six sections, each of which contains a set of Common Data Elements (CDEs):
  - Patient Information
  - Clinical Indication
  - Technical Information
  - Result
  - Syndromic CDEs
  - Feature-based CDEs
- If an imaging finding is consistent with a well-established syndrome, please describe the finding using the Syndromic CDEs section and not the Feature-based CDEs section.
- If an imaging finding is not consistent with a well-established syndrome, or if there is uncertainty about the interpretation of the finding, please describe the finding using the Feature-based CDEs section and not the Syndromic CDEs section.
- With respect to the chronicity of imaging findings, please use your clinical judgment. Acute findings are those which are thought to be recent. If a finding has both acute and chronic components, please indicate both.
- All CDEs in this CRF are considered Core and as such are required for completion of the CRF.
- All CDEs in this CRF are labeled with an asterisk (*) for consistency with the version of this CRF that contains the Core and Supplemental CDEs.

## PATIENT INFORMATION

1. Study ID number:* ____
2. Date of onset of first symptom of COVID-19 (MM / DD / YYYY):* ____ / ____ / ______
3. Date of onset of first neurological symptom (MM / DD / YYYY):* ____ / ____ / ______
4. Date of first positive test for SARS-CoV-2 (MM / DD / YYYY):* ____ / ____ / ______
5. Date of imaging study (MM / DD / YYYY):* ____ / ____ / ______

## CLINICAL INDICATION

1. Scan purpose (select all that apply):*

Diagnostic

Post-treatment

Monitoring

Follow-up

Other, specify: __________

1. Neurological symptoms at time of scan (select all that apply):*

None

Focal deficits

Seizures

Confusion/delirium

Coma/disorder of consciousness

Other: ____________________

## TECHNICAL INFORMATION

1. Imaging modality (select all that apply):*

Non-contrast head CT: CT angiogram neck:

CT venogram head: Postcontrast head CT:

CT angiogram head:

## RESULT*

Normal Abnormal (acute) Abnormal (chronic) Abnormal (acute and chronic) Indeterminate

SYNDROMIC CDEs

Ischemic Stroke

1. Present?:*

Yes  No  Indeterminate

1. Hemorrhagic Transformation:

Present?:*

Yes  No  Indeterminate

1. Leukoaraiosis (i.e. small vessel ischemic disease, white matter hypodensities) on CT:

Present?:*

Yes  No  Indeterminate

Hemorrhage

1. Intraparenchymal hematoma:

Present?:*

Yes  No  Indeterminate

1. Intraventricular hemorrhage (IVH):

Present?*

Yes  No  Indeterminate

1. Subarachnoid hemorrhage (SAH):

Present?:*

Yes  No  Indeterminate

1. Epidural Hematoma:

Present?:*

Yes  No  Indeterminate

1. Subdural Hematoma:

Present?:*

Yes  No  Indeterminate

Vascular Abnormalities

1. Present?:*

Yes  No  Indeterminate

Mass Effect and Midline Shift:

1. Midline shift:

Present?:*

Yes  No  Indeterminate

1. Effacement of basal cisterns?*

Open  Partially effaced  Completely effaced

1. Herniation:

Present?:*

Yes  No  Indeterminate

1. Hydrocephalus:

Present?:*

Yes  No  Indeterminate

Mechanism

1. Presumed association of syndrome with COVID-19:*

Associated

Not associated

Uncertain

## FEATURE-BASED CDEs

1) Hypodensity not previously documented in syndromic CDE section

Present?:*

Yes  No  Indeterminate

2) Hyperdensity not previously documented in syndromic CDE section

Present?:*

Yes  No  Indeterminate

3) Enhancement not documented in syndromic CDE section

Present?:*

Yes  No  Indeterminate

4) Arterial abnormality not previously documented in Syndromic CDE section:

Present?:*

Yes  No  Indeterminate

Mechanism

1. Presumed etiolog(ies) of feature-based imaging finding(s) (check all that apply):*

Hypoxia

Hypoxic-ischemic injury

Traumatic brain injury

Inflammation/encephalitis

Related to extracorporeal membrane oxygenation (ECMO)

Hypoglycemia

Abscess

Tumor

Seizure

Other: _________________

1. Presumed association of feature-based finding(s) with COVID-19:*

Associated

Not associated

Uncertain
