# Supplementary material for: Common Data Elements for COVID-19 Neuroimaging: A GCS-NeuroCOVID Proposal
Source: Neurocrit Care. 2021 Feb 11;34(2):365–70. doi: 10.1007/s12028-021-01192-6 (PMC7878171; doi:10.1007/s12028-021-01192-6)
Supplement: Supplementary file 8 — Supplementary material 8 (DOCX 23 kb) [file 12028_2021_1192_MOESM8_ESM.docx]

## User Guide

- This Case Report Form (CRF) is designed to capture the broad spectrum of imaging findings detected by magnetic resonance (MR) perfusion in patients with COVID-19.
- The CRF is organized into four sections, each of which contains a set of Common Data Elements (CDEs):
  - Patient Information
  - Clinical Indication
  - Result
  - Feature-based CDEs
- With respect to the chronicity of imaging findings, please use your clinical judgment. Acute findings are those which are thought to be recent. If a finding has both acute and chronic components, please indicate both.
- All CDEs in this CRF are considered Core and as such are required for completion of the CRF.
- All CDEs in this CRF are labeled with an asterisk (*) for consistency with the version of this CRF that contains the Core and Supplemental CDEs.

## PATIENT INFORMATION

1. Study ID number:* ____
2. Date of onset of first symptom of COVID-19 (MM / DD / YYYY):* ____ / ____ / ______
3. Date of onset of first neurological symptom (MM / DD / YYYY):* ____ / ____ / ______
4. Date of first positive test for SARS-CoV-2 (MM / DD / YYYY):* ____ / ____ / ______
5. Date of imaging study (MM / DD / YYYY):* ____ / ____ / ______

## CLINICAL INDICATION

1. Scan purpose (select all that apply):*

Diagnostic

Post-treatment

Monitoring

Follow-up

Other, specify: __________

1. Neurological symptoms at time of scan (select all that apply):*

None

Focal deficits

Seizures

Confusion/delirium

Coma/disorder of consciousness

Other: ____________________

## RESULT*

Normal Abnormal (acute) Abnormal (chronic)

Abnormal (acute and chronic) Indeterminate

FEATURE-BASED CDEs

Mechanism

1. Presumed etiolog(ies) of feature-based imaging finding(s) (check all that apply):*

Hypoxia

Hypoxic-ischemic injury

Traumatic brain injury

Inflammation/encephalitis

Related to extracorporeal membrane oxygenation (ECMO)

Hypoglycemia

Abscess

Tumor

Seizure

Other: _________________

1. Presumed association of feature-based finding(s) with COVID-19:*

Associated

Not associated

Uncertain
