# Supplementary material for: Common Data Elements for COVID-19 Neuroimaging: A GCS-NeuroCOVID Proposal
Source: Neurocrit Care. 2021 Feb 11;34(2):365–70. doi: 10.1007/s12028-021-01192-6 (PMC7878171; doi:10.1007/s12028-021-01192-6)
Supplement: Supplementary file 9 — Supplementary material 9 (DOCX 24 kb) [file 12028_2021_1192_MOESM9_ESM.docx]

## USER GUIDE

- This Case Report Form (CRF) is designed to capture the broad spectrum of imaging findings detected by magnetic resonance (MR) spectroscopy in patients with COVID-19.
- The CRF is organized into five sections, each of which contains a set of Common Data Elements (CDEs):
  - Patient Information
  - Clinical Indication
  - Technical Information
  - Result
  - Feature-based CDEs
- With respect to the laterality of imaging findings, please report the findings in anatomic convention, not radiologic convention.
- With respect to the chronicity of imaging findings, please use your clinical judgment. Acute findings are those which are thought to be recent. If a finding has both acute and chronic components, please indicate both.
- Core CDEs, which are required for completion of this CRF, are indicated with an asterisk (*). All other CDEs are Supplemental.

## PATIENT INFORMATION

1. Study ID number:* ____
2. Date of onset of first symptom of COVID-19 (MM / DD / YYYY):* ____ / ____ / ______
3. Date of onset of first neurological symptom (MM / DD / YYYY):* ____ / ____ / ______
4. Date of first positive test for SARS-CoV-2 (MM / DD / YYYY):* ____ / ____ / ______
5. Date of imaging study (MM / DD / YYYY):* ____ / ____ / ______

## CLINICAL INDICATION

1. Scan purpose (select all that apply):*

Diagnostic

Post-treatment

Monitoring

Follow-up

Other, specify: __________

1. Neurological symptoms at time of scan (select all that apply):*

None

Focal deficits

Seizures

Confusion/delirium

Coma/disorder of consciousness

Other: ____________________

TECHNICAL INFORMATION

1. Name of the scanner manufacturer:

GE  Siemens  Philips  Other:

1. Name of the scanner type: ________________
2. Magnetic Field Strength of Scanner Used:

1.5 T  3.0 T  4.0 T  7.0 T  Other:

1. Body part scanned:*

Brain Spinal cord

1. Nucleus

^1^H  ^31^P  Other:

1. RF receiver coil:

Surface coil  Head coil  Body coil

1. Volume localization technique

PRESS  STEAM  Other

1. Number of voxels:

Single voxel (SVS)  Spectroscopic Imaging (SI)-2D  SI-3D

1. Spectroscopy sequence parameters
2. SVS
3. Anatomical location of voxel: __________
4. Voxel volume: _____ mm^3^
5. SI
6. Anatomical location of slab/ROI;
7. Slice/slab: thickness (mm); x,y dimensions (mm)
8. Matrix size: In-plane: x;y (+ z-direction for 3D-SI)
9. SI voxel volume
10. Water suppression scheme:

CHESS  Other  Not relevant – not ^1^H MRS

1. Number of averages: ________
2. Acquisition time: ______ (minutes)
3. TR: ______ (msec)
4. TE: ______ (msec)
5. Processing and quantitation
6. Metabolite concentration reported in:

Absolute value  Institutional unit  Ratio

1. Spectral fit software used:

LCM  jMRUI  Other

## RESULT*

Normal Abnormal (acute) Abnormal (chronic)

Abnormal (acute and chronic) Indeterminate

## FEATURE-BASED CDEs

1. If ^1^H MRS and abnormal, report abnormality for following metabolites [or metabolite ratios]:*

N-acetyl- aspartate: __________

Choline: __________

Creatine: __________

Myo-inositol: __________

Lactate: __________

Glutamine/glutamate (Glx): __________

Other: __________

Mechanism

1. Presumed etiolog(ies) of feature-based imaging finding(s) (check all that apply):*

Hypoxia

Hypoxic-ischemic injury

Traumatic brain injury

Inflammation/encephalitis

Related to extracorporeal membrane oxygenation (ECMO)

Hypoglycemia

Abscess

Tumor

Seizure

Other: _________________

1. Presumed association of feature-based finding(s) with COVID-19:*

Associated

Not associated

Uncertain
